# PredPhos: an ensemble framework for structure-based prediction of phosphorylation sites

**DOI:** 10.1186/s40709-016-0042-y

**Published:** 2016-07-04

**Authors:** Yong Gao, Weilin Hao, Jing Gu, Diwei Liu, Chao Fan, Zhigang Chen, Lei Deng

**Affiliations:** School of Software, Central South University, No. 22 Shaoshan South RD., Changsha, 410075 China; School of Electronics Engineering and Computer Science, Peking University, No. 5 Yiheyuan Road, Beijing, 100871 China; Shanghai Key Laboratory of Intelligent Information Processing, No. 220 Handan Road, Shanghai, 200433 China

**Keywords:** Phosphorylation sites, Ensemble learning, Structural neighborhood properties

## Abstract

**Background:**

Post-translational modifications (PTMs) occur on almost all proteins and often strongly affect the functions of modified proteins. Phosphorylation is a crucial PTM mechanism with important regulatory functions in biological systems. Identifying the potential phosphorylation sites of a target protein may increase our understanding of the molecular processes in which it takes part.

**Results:**

In this paper, we propose PredPhos, a computational method that can accurately predict both kinase-specific and non-kinase-specific phosphorylation sites by using optimally selected properties. The optimal combination of features was selected from a set of 153 novel structural neighborhood properties by a two-step feature selection method consisting of a random forest algorithm and a sequential backward elimination method. To overcome the imbalanced problem, we adopt an ensemble method, which combines bootstrap resampling technique, support vector machine-based fusion classifiers and majority voting strategy. We evaluate the proposed method using both tenfold cross validation and independent test. Results show that our method achieves a significant improvement on the prediction performance for both kinase-specific and non-kinase-specific phosphorylation sites.

**Conclusions:**

The experimental results demonstrate that the proposed method is quite effective in predicting phosphorylation sites. Promising results are derived from the new structural neighborhood properties, the novel way of feature selection, as well as the ensemble method.

## Background

Protein phosphorylation is one of the most prevailing post-translational modifications [1], playing a significant role in regulating almost every cellular process, including transcription [2], translation [3] and signal transductions [4], etc. It is estimated that around 30–50 % of proteins in eukaryotic cells are phosphorylated and abnormal phosphorylation is now recognized as a cause of human disease, especially cancer [5]. Considering its prominent role in biochemistry, researches in identifying phosphorylation sites are booming in recent years.

Historically, the experimental methods of phosphorylation site annotation have undergone several stages from low-throughput proteomics based on site-directed mutagenesis to high-throughput biological technique [6–12] with the advent of mass spectrometry. Providing a number of verified phosphorylation sites, experimental identification is pivotal in understanding the mechanism of phosphorylation dynamic and provides the guidance in biomedical drug design.

Several databases have been established to store annotated phosphorylation sites. Swiss-Prot [13] is a widely used protein sequence and knowledge database, which provides plentiful information about the post-translational modification. PhosphoBase [14] is another database which specifically stores experimentally verified phosphorylation sites, collected from Swiss-Prot and PIR protein database, literature studies and experiments. Phospho.ELM [15] contains 8718 substrate proteins covering 3370 tyrosine, 31,754 serine and 7449 threonine instances. Information about the phosphorylated proteins and the exact position of known phosphorylated instances, the kinases responsible for the modification and links to bibliographic references can be gained from the entries. PhosphoPep [16] contains 12,756 assigned phosphorylation sites identified in *Drosophila melanogaster* Kc167 cells. Four species of phosphoproteome data are included in PhosphoPep, which are yeast (*Saccharomyces cerevisiae*), worm (*Caenorhabditis elegans*), fly (*D. melanogaster*) and human (*Homo sapiens*), respectively, and a novel function was implemented to analyze the conservation of the identified phosphorylation sites across species. PHOSIDA [17] is a database aims to manage post-translational modification sites of various species, including human, mouse, fly, worm and yeast proteins. Also, PHOSIDA provides a wide range of analysis tools. Under the demand for analyzing the structural features of experimentally verified phosphorylation sites, Phospho3D [18] was launched for storing information retrieved from Phospho.ELM and was enriched with structural information and annotation at the residue level.

Given a long list of candidate phosphorylation sites in protein of interest, efforts to verify all of them by time-consuming and resource-intensive biological techniques remain challenging [19]. Alternatively, computational approaches have become increasingly popular. Up to now, there have been around 40 phosphorylation site prediction tools being established, varying from one tool to another with respect to several particular attributes, including dataset construction, feature selection, training system design and so on. The prediction tools can be grouped into two categories: kinase-specific and non-kinase-specific tools. A kinase-specific prediction program requires as input both a protein sequence and the type of a kinase, and produces some measure of the likelihood that each S/T/Y residue in the sequence is phosphorylated by the chosen kinase. In contrast, a non-kinase-specific prediction tool requires only a protein sequence as input, and reports the likelihood that each S/T/Y residue is phosphorylated by any possible kinase. DISPHOS [20] and NetPhos [21] are two typical non-specific predictors. DISPHOS investigated more than 1500 experimentally determined phosphorylation sites in eukaryotic proteins deriving from Swiss-Prot combined with PhosphoBase. Position-specific amino acid frequencies and disorder information are two of its crucial features. As to another non-specific phosphorylation site predictor called NetPhos, 584 serine sites, 108 threonine sites and 210 tyrosine sites were extracted mainly from PhosphoBase. An artificial neural network method is used to predict phosphorylation site with both sequence and structure information. NetPhosK [22] is a kinase-specific phosphorylation site predictor selecting six serine/threonine kinases mainly from PhosphoBase, which are PKA, PKC, PKG, cdc2, CK-2 and CaM-II. Another state-of-art kinase-specific prediction tool named KinasePhos [23] identifies phosphorylation sites based on Hidden Markov Model (HMM) in KinasePhos 1.0 and support vector machine (SVM) in KinasePhos 2.0, using experimentally validated phosphorylation sites from both PhosphoBase and Swiss-Prot. GPS [24] gained totally more than 2000 phosphorylation sites mainly from Phospho.ELM and could predict more than 70 kinds of kinase-specific phosphorylation sites. Based on similar data set of GPS, PPSP [25] predicts kinase-specific phosphorylation sites for 68 kinds of kinases, implementing an algorithm of Bayesian decision theory (BDT). In addition, PPSP can be used for many novel kinases, such as TRK, mTOR, SyK, and MET/RON, etc.

Although much progress has been made, there still exist several difficulties which keep phosphorylation site prediction far away to be perfectly solved. Firstly, while one-dimension sequence information is proved to harbor most of the predictive power, recently published analyses pointed out that phosphorylation sites may be closely related with its structural conformations and, furthermore, affected by the specific three-dimensional spatial environment [26]. Secondly, various kinds of features containing either sequence or structural information seems to be too sufficient for a predictor to be trained and a superabundant feature set would reduce calculation efficiency and increase space complexity. Thirdly, and also most importantly, the imbalanced problem exists widely in phosphorylation site prediction because the number of phosphorylation sites of a protein is usually much smaller than that of non-phosphorylation sites. The imbalanced data tends to cause over-fitting and poor performance.

In this paper, we report a novel structure-based computational method, PredPhos, that combines three main sources of information, namely site, Euclidean and Voronoi features describing the properties of either the target residue or the target residue’s structural neighborhood. A set of optimal features were selected from 153 site, Euclidean and Voronoi properties by a two-step feature selection method (Table 1). Also, PredPhos uses a hybrid approach, which incorporates bootstrap resampling technique, SVM-based fusion classifiers and majority voting strategy, to overcome the imbalanced problem. We have benchmarked PredPhos using a set of experimentally verified phosphorylation sites and an independent dataset. Results show that PredPhos significantly outperforms the state of the art methods for both kinase-specific and non-kinase-specific phosphorylation site prediction.

**Table 1:** Performance of the two-step feature selection method

	AUC	Accuracy	Recall	Specificity	CC	F1
CK2						
Optimal features	0.877	0.963	0.433	0.992	0.433	0.429
All features	0.842	0.954	0.350	0.986	0.370	0.366
MAPK						
Optimal features	0.839	0.952	0.483	0.973	0.480	0.480
All features	0.833	0.959	0.400	0.985	0.424	0.423
PKA						
Optimal features	0.858	0.948	0.375	0.980	0.426	0.432
All features	0.846	0.926	0.335	0.959	0.279	0.310
PKC						
Optimal features	0.857	0.952	0.303	0.985	0.396	0.363
All features	0.821	0.948	0.226	0.984	0.282	0.274
SRC						
Optimal features	0.900	0.951	0.558	0.973	0.510	0.499
All features	0.890	0.946	0.241	0.985	0.317	0.294

## Methods

### Datasets

The experimentally verified phosphorylation sites were extracted from Phospho.ELM version 9.0 [15], PhosphoPOINT [27] and PhosphoSitePlus [28]. After removing the redundant sites among these databases, 44,663 phosphoproteins which had at least one phosphorylated site were exacted in the first step. Phosphorylation sites were mapped to the protein entries of Protein Data Bank (PDB) by using Blast [29] with sequence similarity ≥90 %. Redundant PDB sequences were removed with 90 % sequence identity through CD-HIT [30]. 981 phosphoprotein chains and 2404 phosphorylation sites were remained. Negative phosphorylation sites gathered from their respective proteins had to meet three criteria: (1) a potential negative site could not have been reported as a positive site; (2) it had to be within a protein that contained known positive sites; and (3) a negative phosphorylation site had to be solvent-inaccessible. We divided these data into a training set and an independent test set based on the date the phosphorylation sites deposited to the database. Phosphorylation sites deposited before the year of 2008 were used to construct the non-kinase-specific training set, and the remaining sites composed the independent non-kinase-specific test set.

For the kinase-specific evaluation, training sites and test sites in the family of PKA, PKC, CK2, SRC and MAP were selected from the non-kinase-specific training set and independent test set, respectively.

### Evaluation measures

The performance of the proposed prediction method is evaluated using tenfold cross-validation. The training data set is randomly divided into ten subsets with an approximately equal number of residues. For each time, nine subsets are used as training data and the remaining subset is used as test data.

Several widely used measures are adopted in this study, including *sensitivity (recall), specificity, precision, correlation coefficient (*CC*),* F1-*score and* AUC *score.*

These measures are defined as follows:

$$Sensitivity/Recall = {\text{TP}}/\left( {{\text{TP }} + {\text{FN}}} \right);$$

$$Specificity = {\text{TN}}/\left( {{\text{FP}} + {\text{TN}}} \right);$$

$$Precision = {\text{TP}}/\left( {{\text{TP}} + {\text{FP}}} \right);$$

$$CC = \frac{(TP \times TN - FP \times FN)}{{\sqrt {(TP + FN)(TP + FP)(TN + FP)(TN + FN)} }};$$

$${\text{F}}1 = 2 \times Precision \times Recall/(Precision + Recall)$$

Above, the *TP, FP, TN* and *FN* are abbreviations of the number of true positives, the number of false positives, the number of true negatives and the number of false negatives, respectively. The *AUC* score is the normalized area under the ROC curve. The ROC curve is plotted with *TP* as a function of *FP* for various classification thresholds.

### PredPhos framework

**Fig. 1:**
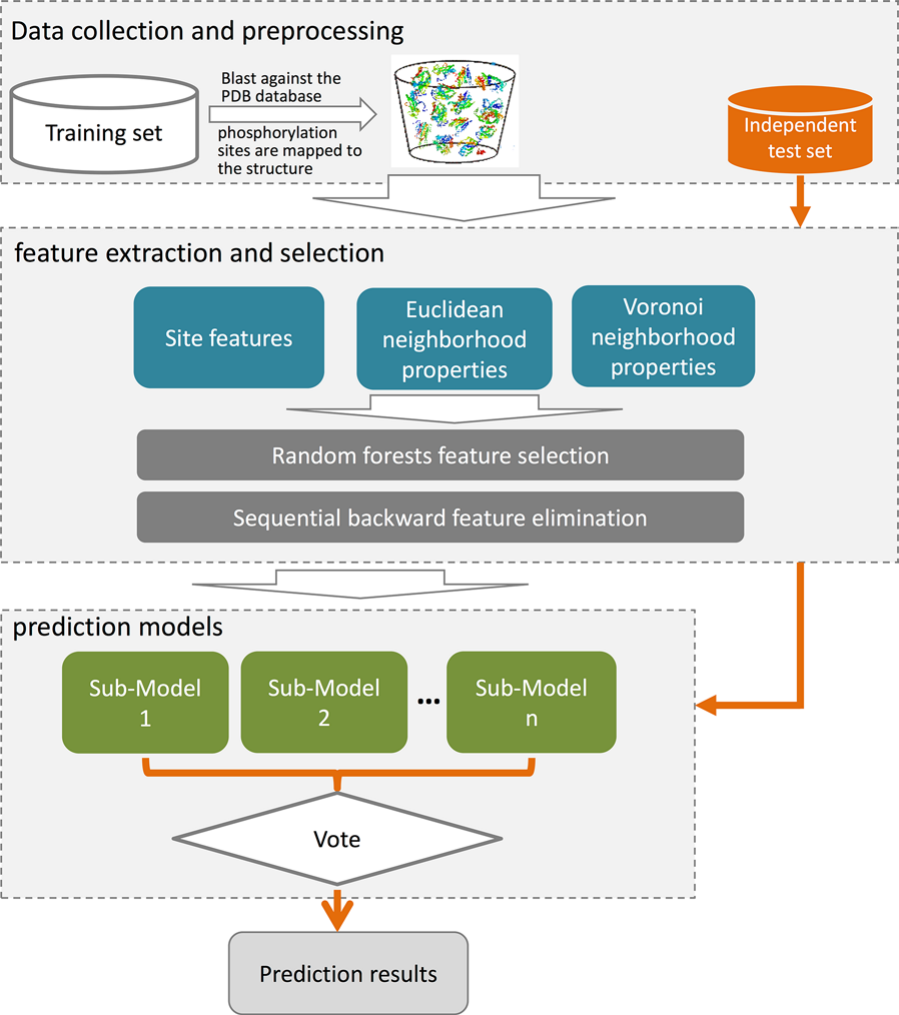
The framework of PredPhos. Phosphorylation sites in the training set were mapped to the protein entries of Protein Data Bank (PDB) by using Blast. We encode each residue using 51 site features, 51 Euclidean neighborhood features and 51 Voronoi neighborhood features. The first step of feature selection is done by a random forest algorithm. Features are ranked in descending order by Z-Scores and the top 80 features are selected. The second step is performed using a wrapper-based feature selection. Features are evaluated by tenfold cross-validation with the SVM algorithm, redundant features are removed by sequential backwards elimination. Finally, an ensemble of n classifiers is built using different subsets, the final result is determined by majority votes among the outputs of the n classifiers

The framework of PredPhos is shown in Fig. 1. The computational approach used by PredPhos consists of three main component processes: (1) data collection and preprocessing: phosphorylation sites of both training and independent test set are mapped to the PDB structures with by using Blast [29] with sequence similarity ≥90 %; (2) feature extraction and selection: extract a wide variety of sequence, structural, and energy features, together with two types of structural neighborhoods, a two-step feature selection process that combines random forest and a sequential backward elimination; and (3) prediction models: ensemble classifiers are built for identifying phosphorylation sites based on the optimally selected features.

### Feature extraction and selection

#### Site features

A large variety of 51 sequence, structural, and energy attributes are selected for the phosphorylation sites classification. Both conventional and new attributes were exploited in this kind of study, including PSSM (20) [29, 31, 32], evolutionary conservation score (1) [33], disorder (6) [34, 35], Solvent accessible area (ASA) (2) [36], pair potential (1) [37], atom and residue contacts (2) [38], Topographical index (1) [39], physicochemical features (6) [40], four-body statistical pseudo-potential (1) [41], local structural entropy (2) [42], side-chain energy (6) [41], Voronoi contacts (2) [43] and structural conservation score (1) [44]. The most interesting features are described below.

### Four-body pseudo-potential

Given unique properties, the Delaunay tessellation [41] is an optimal choice when nearest neighbors should be objectively defined. Based on the Delaunay tessellation of proteins, the four-body pseudo-potential is defined as follows:

1 $$Q_{ijkl}^{\alpha } = \log \left[ {\frac{{f_{ijkl}^{\alpha } }}{{p_{ijkl}^{\alpha } }}} \right]$$

Above, *i, j, k* and *l* represent the residue identities of the four amino acids (20 possibilities) in a Delaunay tetrahedron from the tessellation of the protein. Each residue is represented by a single point located at the centroid of the atoms in its side chain. Also, $f_{ijkl}^{\alpha }$ is the observed frequency of the residue composition (*ijkl)* in a tetrahedron of type a over a set of protein structures, while $p_{ijkl}^{\alpha }$ is the expected random frequency.

### Local structural entropy

Each residue has its unique local structural entropy (LSE) [42], which can be calculated according to the protein sequence. The possibility of each candidate amino acid existing in eight secondary structure types (*α*-helices, *π*-helices, *β*-bridges, extended *β*-sheets, 3_10_-helices, bends, turns and others) defined by DSSP is computed by averaging four sequential sequence windows along the protein sequence. A higher value of LSE indicates this amino acid is more likely to be found in these secondary structures.

In addition, an original attribute named ΔLSE is defined in order to estimate the distinction of LSE score between the wild-type protein and its mutants.

### Side chain energy score

Each given residue of a protein has its own energy score which is originally applied for protein design [41]. A side chain energy score is a linear combination of various energetic terms, including buried hydrophilic solvent accessible surface between the current residue and the rest of the protein, buried hydrophobic solvent accessible surface, atom contact surface area, electrostatic interaction energy, hydrogen bonding energy, and overlap volume.

### Structural conservation score

For a query protein, structural neighbors are obtained by using the structure alignment method-Ska [45]. Contact frequency maps are generated based on the mappings between the neighbor’s surface residues and the surface residues of the query protein [46]. Based on the contact frequency maps, conservation scores of each surface residue is calculated to evaluate degrees of interface conservation.

For all the site features, phosphorylation sites were represented as peptides of length 15, with the phosphorylated residue in the center and seven amino acids on either side. When a particular phosphorylated residue was too close to the beginning or end of the protein to have seven residues on either side, the missing residues were represented by gap (_) characters.

#### Structural neighborhood properties

Most of the conventional features such as physicochemical features, evolutionary conservation, and solvent accessible area describe only the properties of the current site itself, cannot represent the real situation efficiently, and thus are insufficient to predict phosphorylation sites with high accuracy. Here, we develop a new way to calculate two types of structural neighborhood properties using Euclidean distance and Voronoi diagram [47].

The Euclidean neighborhood is a group of residues located within a sphere of 10 Å defined by the minimum Euclidean distances between any heavy atoms of the surrounding residues and any heavy atoms from the central residue. The value of a specific residue-based feature *f* for neighbor *j* with regard to the target residue *i* is defined as

2 $${P_f}(i,j) = \left\{ \begin{array}{l} {\rm{the}}{\mkern 1mu}\, {\rm{value}}\,{\mkern 1mu} {\rm{of}}\,{\mkern 1mu} {\rm{feature}}\,{\mkern 1mu} {\rm{f}}\,{\mkern 1mu} {\rm{for}}\,{\mkern 1mu} {\rm{residue }}\,{\mkern 1mu} {\rm{j}}{\mkern 1mu} \quad {\rm{if}}{\mkern 1mu} \left| {{\rm{i}}{\mkern 1mu} - {\mkern 1mu} {\rm{j}}} \right| \ge 1{\mkern 1mu} \quad {\rm{and}} \quad {\mkern 1mu} {d_{i,j}} \le 10\,{\AA}\\ 0{\mkern 1mu} {\mkern 1mu} \qquad {\rm{otherwise}} \end{array} \right.$$

Above, *d*_*i,j*_ is the minimum Euclidean distance between any heavy atoms of residue *i* and any heavy atoms of residue *j*. The Euclidean neighborhood property of target residue *i* is defined as follows:

3 $$ENP_{f} (i) = \sum\limits_{j = 1}^{n} {P_{f} (i,j)}$$

where *n* is the total number of Euclidean neighbors.

We also use Voronoi diagram/Delaunay triangulation to define neighbor residues in 3D protein structures. For a protein structure, Voronoi tessellation partitions the 3D space into Voronoi polyhedra around individual atoms. Delaunay triangulation is the dual graph of Voronoi diagram, a group of four atoms whose Voronoi polyhedra meet at a common vertex form a unique Delaunay tetrahedra. In the context of Voronoi diagram (Delaunay triangulation), a pair of residues are said to be neighbors when at least one pair of heavy atoms of each residue have a Voronoi facet in common (in the same Delaunay tetrahedra). The definition of neighbors is based on geometric partitioning other than the use of an absolute distance cutoff, and hence is considered to be more robust. Voronoi/Delaunay polyhedra are calculated using the Qhull package that implements the Quickhull algorithm developed by Barber et al. [48]. Figure 1 illustrates an example of Voronoi/Delaunay neighbors (green) of a target residue (red).

Given the target residue *i* and its neighbors {*j* = 1,…,*n*}, for each site feature *f*, a Voronoi/Delaunay neighborhood property is defined as follows:

4 $$VDP_{f} = \sum\limits_{j = 1}^{n} {P_{f} (j)}$$

where *P*_*f*_(*j*) is the value of the site feature *f* for residue *j*.

#### Two-step feature selection

In this paper, we propose a two-step feature selection method, as summarized in Algorithm 1, to select a subset of features that contribute the most in the classification.

In the first step, we assess the feature vector elements using the mean decrease Gini index (MDGI) calculated by the RF package in R [49, 50]. MDGI represents the importance of individual feature vector element for correctly classifying an interface residue into phosphorylation sites and non-phosphorylation sites. The mean MDGI Z-Score of each vector element is defined as

5 $$MDGI\,{\text{Z}} - {\text{Score}}\,= \,\frac{{x_{i} - \bar{x}}}{\sigma }$$

where is the mean MDGI of the *i*-*th* feature, $\bar{x}$ is the mean value of all elements of the feature *x*, and *σ* is the standard deviation (SD). Here, we select the top 80 features.

The second step is performed using a wrapper-based feature selection where features are evaluated by tenfold cross-validation performance with the SVM algorithm, and redundant features are removed by sequential backward elimination (SBE). The SBE scheme sequentially removes features from the whole feature set till an optimal feature subset is obtained. Each removed feature is the one whose removal maximizes the performance of the predictor. The ranking criterion *R*_*c*_(*i*) represents the prediction performance of the predictor, which is built on a subset features exclusive of feature *i*, and is defined as follows:

6 $$R_{c} (i) = \frac{1}{k}\sum\limits_{j = 1}^{k} {\{ AUC_{j} + Accu_{j} + Sen_{j} + Spe_{j} \} }$$

where *k* is the repeat times of tenfold cross validation; *AUC*_*j*_, *Accu*_*j*_, *Sen*_*j*_ and *Spe*_*j*_ represent the values of AUC score, accuracy, sensitivity and specificity, respectively.



### Prediction models

PredPhos uses an ensemble of *n* classifiers and decision fusion technique on the training datasets. An asymmetric bootstrap resampling approach is adopted to generate subsets. It performs random sampling with replacement only on the majority class so that its size is equal to the number of minority samples, and keeps the entire minority samples in all subsets.

First, the majority class of phosphorylation sites is under-sampled and split into *n* groups by random sampling with replacement, where each group has the same or similar size as the minority class of interaction sites. After the sampling procedure, we obtain *n* new datasets from the set of non-phosphorylation sites. Each of the new dataset and the set of phosphorylation sites are combined into *n* new training datasets. Then, we train *n* sub-models by using the *n* new training datasets as input. Each of these classifiers is a SVM. Here the LIBSVM package 2.8 [1] is used with radial basis function (RBF) as the kernel. Finally, a simple majority voting method is adopted in the fusion procedure, and the final result is determined by majority votes among the outputs of the *n* classifiers.

## Results and discussion

### Selection of optimal features

**Fig. 2:**
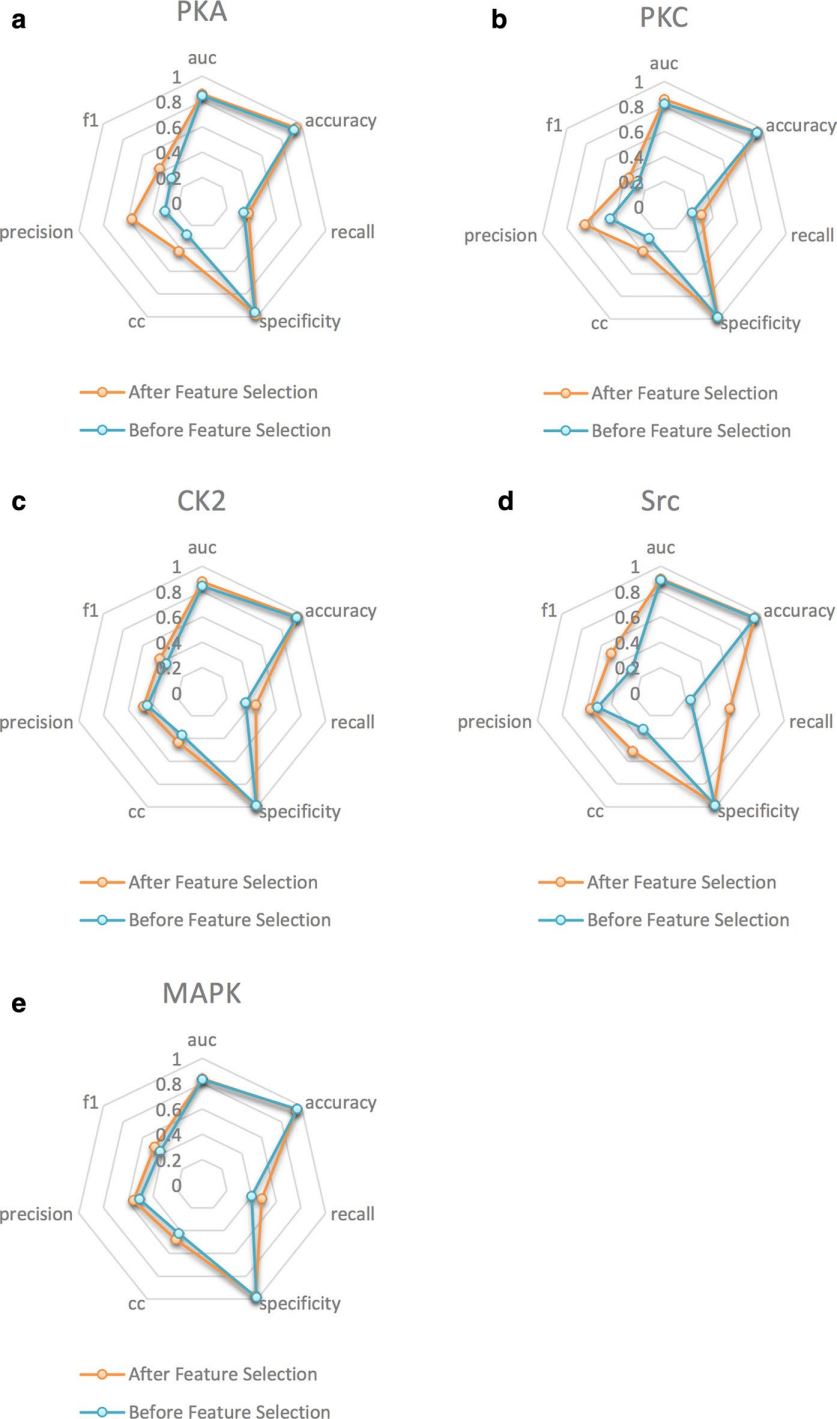
Performance comparison on feature selection and non-feature selection. The performances of PKA, PKC, CK2, SRC and MAPK are shown in **a**, **b**, **c**, **d** and **e**, respectively

We implemented tenfold cross-validation using two distinctive feature sets, namely full set of features (SVM-F) and sub-selected feature set (SVM-Sub). The comparison result is summarized in Table 2 and illustrated in Fig. 2. The performance of each model is measured by six metrics: area under curve (AUC), accuracy (Acc), sensitivity (Sn), specificity (Sp), CC, and F1-score.

**Table 2:** Performance comparison on the independent test dataset

Tools	Kinase family	Sn	Sp	Pre	CC	F1
PPSP	PKA	1.000	0.540	0.096	0.228	0.176
	PKC	0.400	0.527	0.031	−0.028	0.058
	CK2	0.500	0.390	0.038	−0.047	0.071
	SRC	0.538	0.859	0.286	0.304	0.373
	MAPK	0.571	0.380	0.043	−0.021	0.081
Kinasephos	PKA	0.125	0.877	0.048	0.001	0.069
	PKC	0.200	0.863	0.053	0.034	0.083
	CK2	0.500	0.976	0.500	0.476	0.500
	SRC	0.115	0.960	0.231	0.103	0.154
	MAPK	0.571	0.937	0.308	0.381	0.400
NetphosK	PKA	0.375	0.914	0.176	0.204	0.240
	PKC	0.200	0.802	0.037	0.001	0.063
	CK2	0.500	0.805	0.111	0.158	0.182
	SRC	0.038	1.000	1.000	0.187	0.074
	MAPK	0.286	0.979	0.400	0.311	0.333
GPS	PKA	0.500	0.871	0.160	0.222	0.242
	PKC	0.600	0.695	0.070	0.119	0.125
	CK2	0.500	0.854	0.143	0.202	0.222
	SRC	0.462	0.871	0.273	0.265	0.343
	MAPK	0.571	0.789	0.118	0.182	0.195
PredPhos	PKA	0.571	0.779	0.100	0.164	0.170
	PKC	0.824	0.870	0.452	0.544	0.583
	CK2	1.000	0.659	0.176	0.341	0.300
	SRC	0.789	0.802	0.234	0.356	0.361
	MAPK	0.375	0.986	0.600	0.452	0.462

As to accuracy and specificity, SVM-sub performed marginally worse than SVM-F in the family of MAPK (−0.07, −1.2 %) and SRC (+5.3, −1.2 %), but still outperformed in the family of CK2 (+0.9, +0.6 %), PKA (+2.4, +2.2 %) and PKC (+0.4, +0.1 %). It can be observed from Table 2 and Fig. 2 that SVM-sub shows dominant advantages over SVM-F in the other four metrics: AUC, sensitivity, CC, and F1-score for all five families. The improvement derived from the two-step feature selection is so obvious that we can also easily get the intuitive comparison directly from Fig. 2. Concretely, for the family of CK2, after the operation of random forest feature selection based on the full set of 153 features (SVM-F), the value of AUC, sensitivity, CC, and F1-score increased by about 4.2, 23.7, 17 and 17.2 %, respectively. For the family of MAPK, the value of AUC, sensitivity, CC, and F1-score increased by about 0.7, 20.8, 13.2 and 13.5 %, respectively. For the family of PKA, the value of AUC, sensitivity, CC, and F1-score increase by about 1.4, 11.9, 52.7 and 39.4 %, respectively. For the family of PKC, the value of AUC, sensitivity, CC, and F1-score increased by about 4.4, 34, 40 and 32.5 %, respectively. As to the family of SRC, the value of AUC, sensitivity, CC, and F1-score increase by about 1.1, 131.5, 60.9 and 69.7 %, respectively.

Taken the family of PKC as an example, the size of its optimal feature set is 25, although shrank by about 84 % compared with the original size of 153, the prediction performance significantly improved, indicating that our two-step feature selection method can effectively improve the prediction performance with less computational cost and reduce the risk of over-fitting.

**Fig. 3:**
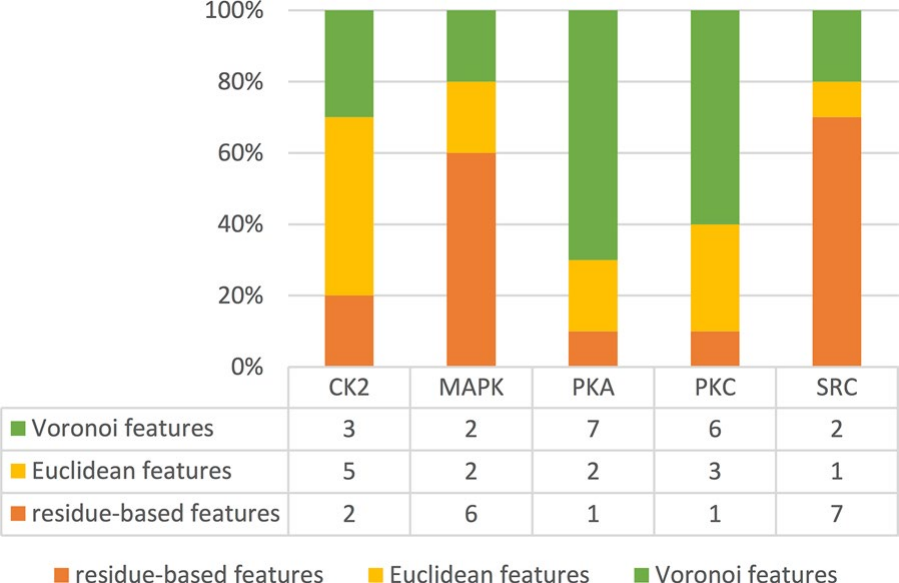
The proportions of residue-based features, Euclidean features and Voronoi features on the top 10 list ranked by the two-step feature selection method for the 5 kinase families

We investigated three types of features including site, Euclidean, and Voronoi features. The proportions of the three types of features on the top 10 list ranked by the two-step feature selection method for 5 families are presented in Fig. 3. From Fig. 3, we can find that for all families except SRC and MAPK, structural neighborhood properties (Euclidean or Voronoi) dominated the top 10 list. To be more specific, CK2 are mainly influenced by Euclidean features while Voronoi features are the most prominent features to PKA and PKC, suggesting that structural neighborhood properties are more predictive for those four families. Opposed to the former 3 families, Fig. 3 indicated that the residue-based features dominated top 10 list for SRC and MAPK. As to MAPK, considering about its large size (112) after feature selection and the poor performance in accuracy and specificity, maybe it suggests that adding its neighborhood properties as feature vectors has few benefit for phosphorylation site prediction.

### Performance comparison with the state of the art approaches on the kinase-specific datasets

In this section, the proposed method (PredPhos) was benchmarked against PPSP, NetPhosK 1.0, KinasePhos 1.0 and GPS 2.1, four widely used kinase-specific predictors on the independent dataset with 171, 136, 43, 274 and 149 phosphorylation sites of family of PKA, PKC, CK2, SRC and MAPK, respectively.

PPSP is implemented in an algorithm of Bayesian decision theory (BDT). KinasePhos is a predictor applying Profile Hidden Markov Model (HMM) for learning to each group of sequences surrounding to the phosphorylation residues. NetPhosK is an artificial neural network method that predicts phosphorylation sites in independent sequences with sensitivity in the range from 69 to 96 %. GPS 2.1 adopted a PK classification established by Manning et al. [5] as the standard rule to cluster the human PKs into a hierarchical structure with four levels, including group, family, subfamily and single PK. To have a performance comparison, we submitted the substrate sequence into the above tools for prediction. KinasePhos has three cut-off values for prediction specificity, as 90, 95 and 100 %. As for PPSP, NetPhosK and GPS, we also adopt different parameters to perform. We choose the best results with different parameters of other four tools to compare with PredPhos. Comparison results of the independent test are presented in Table 2.

It should be noted that without available training methods of most tools, it is nearly an impossible task to compare our predictor with the rest by running cross-validations. Benchmark test is an alternative solution to test and compare our method with others by using the same test set. However, unfair comparison may generate if our test data are included in the training set of other tools, and thus leading a fake high performance of other existing ones and underestimation of ours.

High sensitivity is beneficial when predicting phosphorylation sites in a single protein because, in wet-bench studies, experimental biologists may select some candidates from the predicted sites for further experimental design. However, a method with a high specificity is useful for whole-genome annotation. According to Table 2, the Sn values of PredPhos for the families of PKC, CK2 and SRC were 0.824, 1.000, and 0.789, respectively. PredPhos outperformed all the predictors with high Sn values of the most kinase families. Although all MCC values were not very high, the MCC values of the PredPhos results were also the best ones among other predictors. For the family of PKC, PredPhos has the highest recall (0.824), specificity (0.870), precision (0.452), mcc (0.544) and F1-score (0.583). As shown in Table 2, for the family of MAPK, although having a lower recall (0.375) than GPS (0.571), PredPhos did make a better balance between the positive dataset and negative dataset, and thus, acquired an outperformance in comprehensive strength (the sum of recall (0.375), specificity (0.986), cc (0.600) and F1-score (0.462)) compared with other prediction tools. Note that for SRC, GPS only considers about Y, while S, T and Y are all taken into account in our method, which may lead to a less-explicate prediction result compared with GPS. In any case, the prediction performance of our method is at least comparable with other kinase-specific prediction tools.

### Performance comparison on the non-kinase-specific dataset

To further evaluate the performance of the proposed PredPhos, a widely used non-kinase-specific prediction method, Netphos [21], is evaluated on the independent test set. Netphos used artificial neural networks with both sequence-based and structural-based features. We can see that our PredPhos approach substantially outperforms the Netphos method in six performance metrics (accuracy, recall, specificity, precision, CC and F1 score) (Table 3).

**Table 3:** Performance comparison on the non-kinase-specific dataset

Methods	Accuracy	Recall	Specificity	Precision	CC	F1
Netphos	0.66	0.51	0.68	0.14	0.11	0.21
PredPhos	0.77	0.60	0.82	0.38	0.23	0.45

## Conclusions

In this work, we presented a novel phosphorylation site prediction approach. Experimental results revealed that the proposed method outperformed most existing kinase-specific and non-kinase-specific prediction methods. Three key factors are responsible for our success. First, the wide exploitation of heterogeneous information, i.e. sequence-based, structure-based and energetic features, together with two types of structural neighborhood (Euclidian and Voronoi), provides more important clues for phosphorylation identification. A total of 153 features, including 51 site properties, 51 Euclidian neighborhood properties and 51 Voronoi neighborhood properties, have been investigated. Second, significant lower computational cost and lower risk of over-fitting was achieved by a two-step feature selection. Third, the ensemble classifiers with resampling technique alleviated the imbalanced problem and improved the prediction accuracy. A limitation of structure-based phosphorylation site prediction is that, proteins without structures can’t be predicted well. However, reliable homology models of a large number of sequences can be generated on the residue level, the overall structural coverage of proteins has increased to 40 % [51].

As for the future work, major existing phosphorylation site prediction methods, including NetPhos and GPS, are considered to be integrated into the PredPhos method to further improve the prediction performance by using Bayesian networks.
